# The Choice of Hip Arthroplasty: HRA or THA? Revealed by Meta‐Analysis

**DOI:** 10.1111/os.70019

**Published:** 2025-03-16

**Authors:** Xiao Fan, Yimin Zhou, Tianyu Zhou, Justin P. Cobb, Tengbo Yu

**Affiliations:** ^1^ Department of Orthopaedics Qingdao Municipal Hospital Qingdao China; ^2^ MSk Laboratory, Department of Surgery and Cancer, Faculty of Medicine Imperial College London London UK; ^3^ Dongzhimen Hospital Beijing University of Traditional Chinese Medicine Beigjing China

**Keywords:** clinical efficacy, hip arthrosis, hip resurfacing arthroplasty (HRA), meta‐analysis, safety, systematic review, total hip arthroplasty (THA)

## Abstract

**Background:**

Total hip arthroplasty (THA) is the gold standard for end‐stage hip arthrosis, while hip resurfacing arthroplasty (HRA) is considered a more bone‐conserving alternative. This meta‐analysis aimed to compare the safety and clinical efficacy of HRA and THA.

**Methods:**

The databases of PubMed, EMBASE, Cochrane Library, and CNKI were searched for RCTs comparing HRA and THA in treating hip arthrosis from database initiation to April 2024. Inclusion and exclusion criteria were defined, and data extraction, quality evaluation, and risk bias assessment were performed. A meta‐analysis was conducted using appropriate RevManv5.4 and Stata v14.0 software.

**Results:**

Twenty RCTs from six countries were included. HRA had a similar revision rate, function scores (WOMAC, HSS, OHS, UCLA activity score, EQ‐D, EQ‐5D VAS), and blood levels of cobalt and chromium compared to THA. HRA had fewer complications and less blood loss but required more operating time.

**Conclusions:**

HRA is a safe and effective alternative to THA, with similar revision and functional outcomes, less blood loss, and is particularly suitable for young male patients with a demand for high‐level activities.

## Graphical abstract

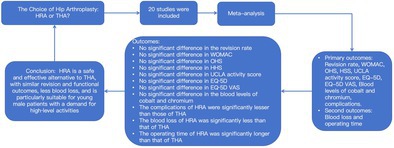

## 1 Introduction

Despite complications such as dislocation and periprosthetic fractures, total hip arthroplasty (THA) has always been considered the gold standard treatment for patients with end‐stage hip arthrosis, including osteoarthrosis, developmental dysplasia of the hip (DDH), and avascular necrosis of the femoral head (ANFH) [1]. Recently, advancements in materials, design, and surgical technique have led to the gradual recognition of hip resurfacing arthroplasty (HRA) as a more bone‐conserving alternative to THA, especially suitable for young and active patients with end‐stage hip arthrosis [2, 3]. The Birmingham HRA system, a classical prosthesis of HRA, has demonstrated superior 10‐year survivorship with acceptable functional outcomes and low rates of complications.

Study has been shown [4] that both THA and HRA offer excellent clinical outcomes for patients with hip arthrosis. While HRA is particularly beneficial for young people, it also provides comparable pain relief and improves hip function for other patients, restoring biomechanics characteristic of the hip and facilitating an earlier return to sports activities [5, 6]. Moreover, some researchers have found that, compared to THA, HRA allows patients to resume sports activities sooner and at higher activity levels. However, concerns [7, 8] have been raised about HRA, including a higher risk of femoral neck fractures and adverse reactions to metal debris (ARMD), among others. To date [9], the incidence of femoral neck fractures, prosthesis loosening or cracking, and ARMD is about 39%, 29%, and 6% respectively. Besides, HRA has a low fault tolerance, which makes it a technical challenge for surgeons.

Currently, there is no clear answer regarding the clinical safety and effectiveness of HRA in treating end‐stage hip arthrosis with high‐level evidence. Meanwhile, there is an undecided debate about whether THA or HRA is the better choice for patients with arthrosis. This study aims to resolve the ongoing debate between THA and HRA through a meta‐analysis and systematic review of their safety and clinical efficacy, providing evidence‐based medical evidence to guide the clinical application and promotion of HRA in treating end‐stage hip arthrosis, which is significantly helpful for surgeons to choose optimal treatments for end‐stage hip arthrosis.

## 2 Materials and Methods

### 2.1 Search Strategy

**FIGURE 1:**
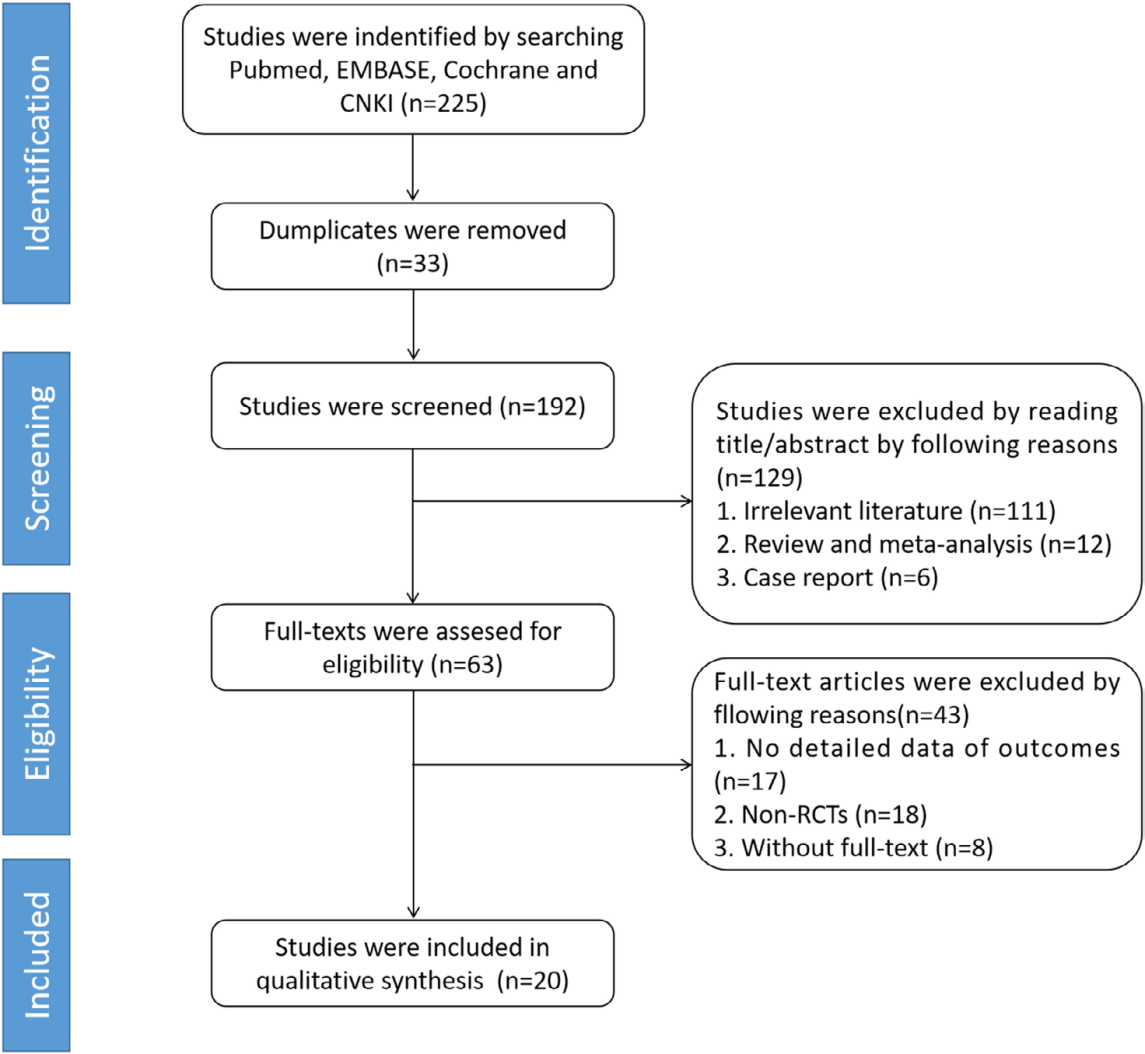
Flow chart of document retrieval process.

The databases of PubMed, EMBASE, Cochrane Library, and CNKI were searched by computer for the randomized controlled trials comparing HRA and THA on treating hip arthrosis, which were published between each database initiation and April 2024, without language restriction. The MeSH, such as hip arthrosis, hip arthroplasty, hip replacement, Randomized Controlled Trial, and Emtree, like hip osteoarthritis, ANFH, DDH, THA, total hip replacement, THA, hip HRA, hip resurfacing replacement, and HRA, were searched for related studies (Figure 1).

### 2.2 Inclusion and Exclusion Criteria

#### 2.2.1 Inclusion Criteria

(i) Randomized controlled trials of patients with hip arthrosis. (ii) Patients diagnosed as hip arthrosis, including hip osteoarthrosis, traumatic arthritis, DDH, ANFH, cam hip, and so forth. (iii) Studies compared HRA and THA. (iv) Outcomes reported as follows: blood loss, operating time, revision rate, Western Ontario and McMaster Universities Osteoarthritis Index (WOMAC), Oxford Hip Score (OHS), Hip Harris Score (HHS), University of California at Los Angeles (UCLA) activity score, EuroQol 5D (EQ‐5D), EQ‐5D VAS, blood levels of cobalt and chromium, complications.

#### 2.2.2 Exclusion Criteria

(i) Nonhuman studies. (ii) Review, meta‐analysis, and case report. (iii) Patients accompanied by other hip diseases or other joint dysfunctions. (iv) Without full text.

### 2.3 Study Selection and Data Extraction

Two researchers (X.F., Y.Z.) screened the titles and abstracts of identified studies independently. If a study met the inclusion criteria, it would be included, and the full text would be reviewed by the two researchers for further analysis independently.

Two researchers (X.F., Y.Z.) independently extracted the data of the included studies as follows: first author, year of publication, age of patients, gender ratio, diagnosis, interventions, follow‐up time, blood loss, operating time, revision rate, WOMAC, OHS, HSS, UCLA activity score, EQ‐5D, EQ‐5D VAS, blood levels of cobalt and chromium, and complications. If there were some divergences about the extracted data between the two researchers, the third researcher made a final decision.

### 2.4 Literature Quality Evaluation and Risk Bias Assessment

Two researchers assessed the quality and risk of bias of the included studies according to the Cochrane risk of bias assessment tool [10] and any disagreement was discussed and resolved by the third researcher.

### 2.5 Outcomes

In the study, the primary outcomes include revision rate, WOMAC, OHS, HSS, UCLA activity score, EQ‐5D, EQ‐5D VAS, blood levels of cobalt and chromium, and complications, and the secondary outcomes include blood loss and operating time.

### 2.6 Statistical Analysis

These data were analyzed using RevManv5.4 and Stata v14.0 software. Disomy and continuous variables were assessed using risk ratio (OR) and mean difference (MD) with 95% confidence intervals (CI) for weighted MD, respectively. Cochran's *Q* and I2 tests were used to determine heterogeneity. When the *I* ^2^ > 50% or the *Q* statistic *p*‐value is < 0.05, the random‐effects model is used; otherwise, the fixed‐effect model is used. Funnel charts were used to investigate occurrence bias.

## 3 Results

### 3.1 Literature Selection

A total of 225 studies were initially identified by database searching. After removing duplications (*n* = 33), reviews and meta‐analyses (*n* = 12), case reports (*n* = 6), non‐RCTs (*n* = 18), studies without detailed data (*n* = 17), studies without full text (*n* = 8), and other unrelated studies (*n* = 111), a total of 20 studies were included finally.

### 3.2 Study Characteristics

Among the included 20 studies, there were 4 studies from the Netherlands, 8 studies from Canada, 3 studies from Denmark, 3 studies from the United Kingdom, 1 study from Greece, and 3 studies from China (Table 1).

**TABLE 1:** Characteristics of 20 included studies.

Study	Group	*n*, M/F	Age (years)	Diagnose (OA/AVN/other)	Height (m)	BMI (kg/m^2^)	Follow‐up (years)	Outcomes
Bisseling (2015)	HRA THA	38, 21/17 33, 21/12	57.5 (50–61) 59.2 (51–61)	35/1/2 31/0/2	NR	26.1 (3.1) 28 (5.1)	≥ 3 ≥ 3	①⑨⑪
Costa (2012)	HRA THA	60, 38/22 66, 36/30	56.3 (7.3) 56.6 (6.6)	58/0/2 61/0/5	NR	28.6 (6.3) 28.7 (4.6)	≥ 3 ≥ 3	③⑥⑦⑨
Costa (2018)	HRA THA	60, 36/24 62, 35/27	56.5 (6.9) 56.7 (7.0)	NR	NR	28.4 (6.2) 28.9 (4.8)	≥ 3 ≥ 3	①③⑥⑦⑨⑩⑪
Gerhardt (2018)	HRA THA	38, 21/17 33, 21/12	54.4 (9.5) 56.5 (7.3)	35/1/2 31/0/2	NR	26.1 (3.1) 28 (5.1)	≥ 3 ≥ 3	①⑨
Gerhardt (2019)	HRA THA	16, 11/5 9, 8/1	52 (10) 57 (8)	NR	1.77 1.80	26 (3) 28 (5)	≥ 5 ≥ 5	③⑦⑪
Hersnaes (2021)	HRA THA	36, 26/10 39, 26/13	59.4 (51.2–63.58) 61.9 (56.5–63.2)	NR	NR	27.45 28.4	≥ 5 ≥ 5	①⑨
Jeannette (2013)	HRA THA	20, 12/8 34, 24/10	57 (54–61) 56 (52–62)	NR	NR	28 (24–31) 27 (25–29)	≥ 2 ≥ 2	②⑤⑥⑨⑩⑪
Karampinas (2014)	HRA THA	15, 7/8 16, 11/5	50.47 (9.68) 56 (52–62)	11/0/4 11/0/5	NR	31 (4.14) 31.6 (3.71)	≥ 2 ≥ 2	②⑤
Konan (2021)	HRA THA	48, 43/5 56, 50/6	51.5 52	NR	NR	28.3 28.2	≥ 8 ≥ 8	②③⑤
Kostretzis (2021)	HRA THA	24, 14/10 24, 15/9	50 (7.1) 50 (7.8)	18/1/5 19/2/3	NR	28 (5.9) 28 (4.1)	≥ 12 ≥ 12	①②⑤⑧⑨
Lavigne (2009)	HRA THA	24, 14/10 24, 15/9	49.6 (38–63) 49.8 (33–62)	NR	NR	27.9 (20.2–36.9) 27.8 (20.2–35.6)	≥ 1 ≥ 1	②⑤⑨
Ran (2018)	HRA THA	28, 19/9 40, 28/12	43 (23–64) 47 (22–64)	7/18/3 5/32/3	NR	21.5 (17.8–25.7) 21.8 (17.2–26.2)	≥ 5 ≥ 5	①③④⑤⑨⑩⑪
Smolders (2011)	HRA THA	38, 21/17 33, 21/12	58 (24–65) 59 (37–65)	35/1/2 31/0/2	NR	26 (3.1) 28 (5.1)	≥ 2 ≥ 2	①⑨
Vendittoli (2006)	HRA THA	107, 67/40 103, 70/33	49.1 (23–64) 50.6 (24–65)	81/3/26 78/2/17	1.72 1.72	27.2 (17.6–44.9) 29.6 (17.4–49.1)	≥ 1 ≥ 1	⑨
Vendittoli (2010) (1)	HRA THA	109, 69/40 100, 68/32	49.2 (9.0) 51 (8.6)	84/3/22 78/2/20	1.72 1.72	27 (5.3) 30 (6.1)	≥ 2 ≥ 2	②⑨⑪
Vendittoli (2010) (2)	HRA THA	64, 42/22 53, 33/20	49.3 (25–64) 51 (30–65)	45/1/18 41/1/11	1.71 (1.50–188) 1.71 (1.51–188)	27.1 (17.6–44.9) 29.2 (21.3–48.1)	≥ 2 ≥ 2	①⑨
Vendittoli (2013)	HRA THA	109, 72/37 100, 68/32	49.2 (23–64) 51 (24–65)	80/3/26 80/3/17	1.72 (10.1) 1.72 (9.6)	27 (5.3) 30 (6.1)	≥ 5 ≥ 5	①②⑤⑧⑨
Vendittoli (2020)	HRA THA	104, 67/37 99, 67/32	48.9 (9.0) 50.7 (8.4)	81/3/26 78/2/17	1.71 (151–192) 1.71 (150–195)	26.6 (4.9) 30.0 (6.8)	≥ 15 ≥ 15	①⑤⑨⑩
Wang (2015)	HRA THA	9, 32/8 23, 32/8	35.3 (8.8)	12/6/14	NR	NR	≥ 3 ≥ 3	④
Xiong (2014)	HRA THA	29, 12/17 32, 15/17	47.8 (18–64) 56.1 (25–88)	1/5/23 4/13/15	NR	NR	≥ 0.75 ≥ 0.6	②④⑩⑪

*Note*: Data are mean (standard deviation) unless indicated otherwise.

Abbreviation: NR, not report.

① Revision rate；② WOMAC；③ OHS；④ HHS；⑤ UCLA activity score; ⑥ EQ‐5D; ⑦ EQ‐5D VAS; ⑧ blood levels of cobalt and chromium; ⑨ complications; ⑩ blood loss; ⑪ operating time.

### 3.3 Risk of Bias

**FIGURE 2:**
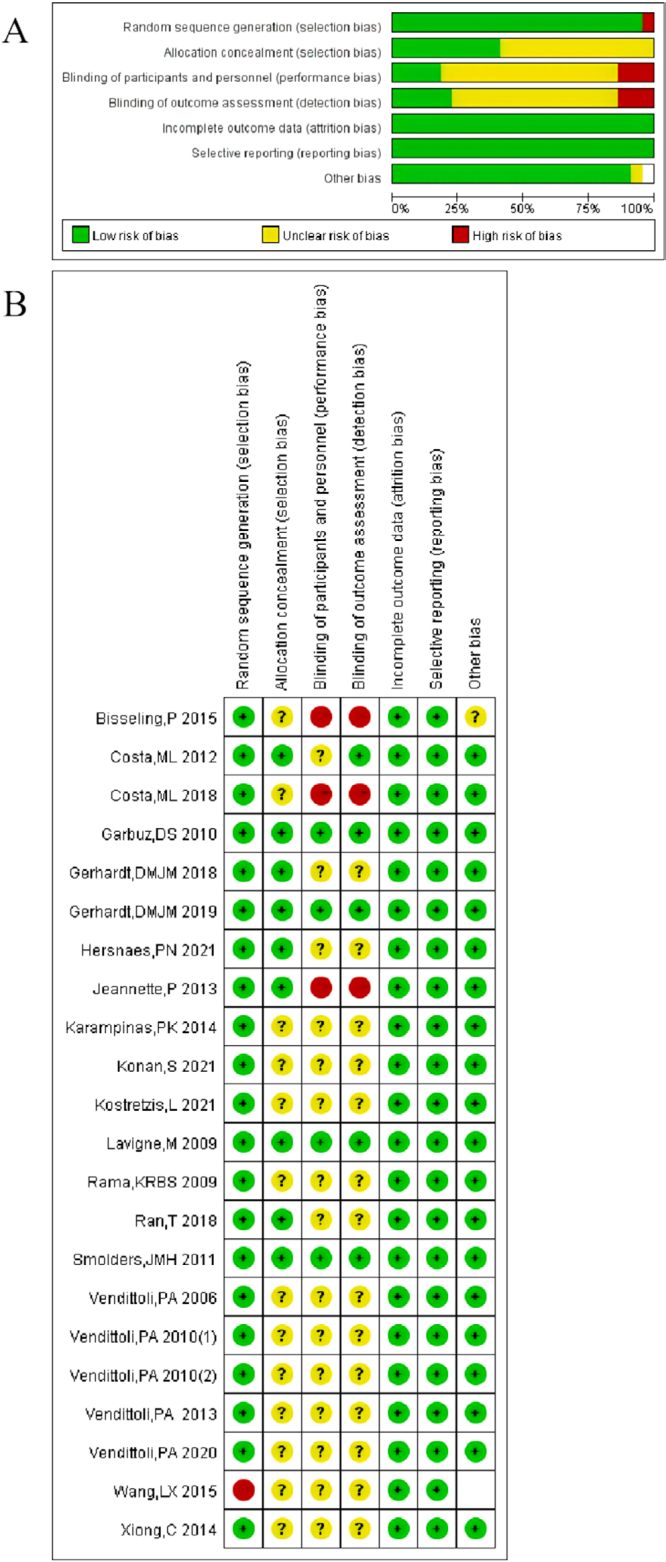
Risk of bias assessment results included in RCTs (Cochrane Manual of Systematic Reviews).

Risk of bias assessment of RCT was assessed using the Cochrane Manual for Systematic Reviews, as shown in Figure 2.

### 3.4 Results of the Meta‐Analysis

#### 3.4.1 Revision Rate

**FIGURE 3:**
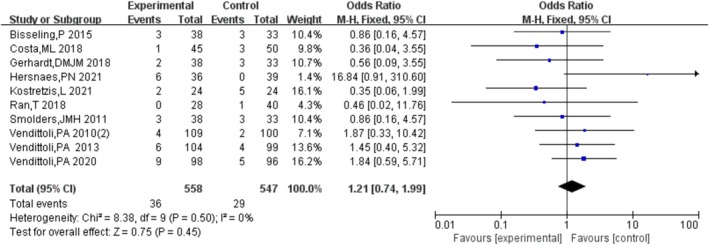
Revision rate.

Ten studies [11, 12, 13, 14, 15, 16, 17, 18, 19, 20] compared the revision rate of HRA and THA, indicating that there was no significant difference in the revision rate between HRA and THA [OR = 1.18, 95% CI = (0.72, 1.94), *p* = 0.52], without heterogeneity (*p* = 0.51, *I* ^2^ = 0%). The results are shown in Figure 3.

#### 3.4.2 WOMAC

**FIGURE 4:**
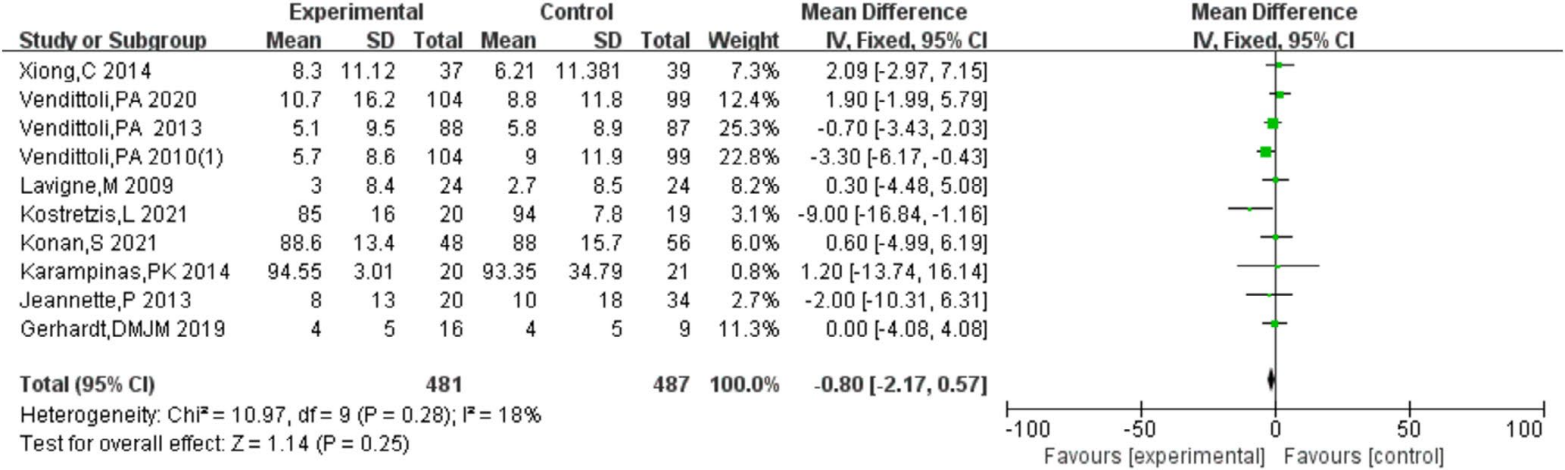
WOMAC.

Ten studies [6, 15, 19, 20, 21, 22, 23, 24, 25, 26] compared the WOMAC of HRA and THA, indicating that there was no significant difference in WOMAC between HRA and THA [MD = −0.97, 95% CI = (−2.33, 0.38), *p* = 0.16], without heterogeneity (*p* = 0.26, *I* ^2^ = 19%). The results are shown in Figure 4.

#### 3.4.3 OHS

**FIGURE 5:**
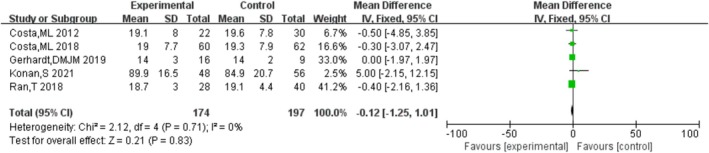
OHS.

Five studies [6, 12, 16, 26, 27] compared the OHS of HRA and THA, indicating that there was no significant difference in OHS between HRA and THA [MD = −0.16, 95% CI = (−1.23, 0.92), *p* = 0.77], without heterogeneity (*p* = 0.71, *I* ^2^ = 0%). The results are shown in Figure 5.

#### 3.4.4 HHS

**FIGURE 6:**
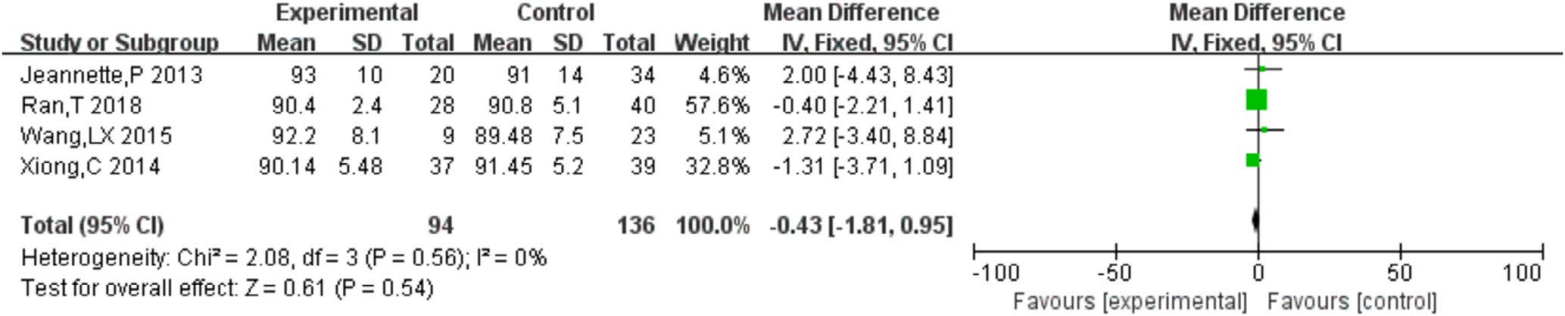
HHS.

Four studies [16, 21, 25, 28] compared the HHS of HRA and THA, indicating that there was no significant difference in HHS between HRA and THA [MD = −0.37, 95% CI = (−1.79, 1.05), *p* = 0.61], without heterogeneity (*p* = 0.58, *I* ^2^ = 0%). The results are shown in Figure 6.

#### 3.4.5 UCLA Activity Score

**FIGURE 7:**
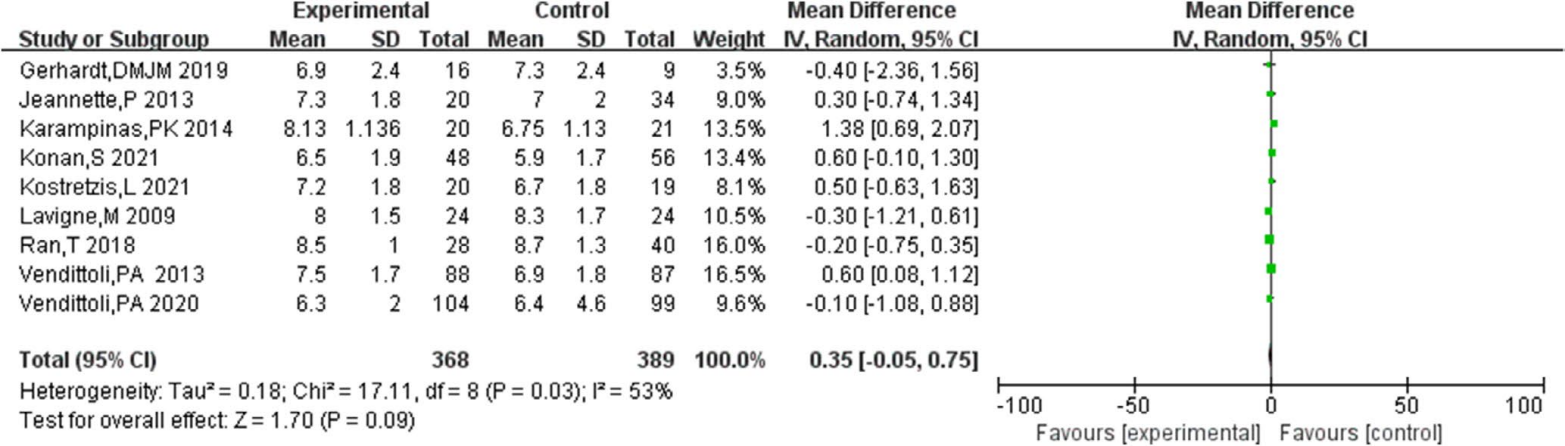
UCLA activity score.

Nine studies [6, 15, 16, 19, 20, 23, 24, 25, 26] compared the UCLA activity score of HRA and THA, with moderate heterogeneity (*p* = 0.05, *I* ^2^ = 48%). The results showed that there was no significant difference in UCLA activity score between HRA and THA [MD = 0.34, 95% CI = (−0.04, 0.71), *p* = 0.08]. The results are shown in Figure 7.

#### 3.4.6 EQ‐5D

**FIGURE 8:**
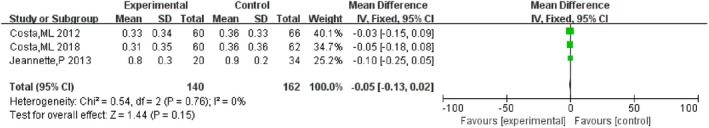
EQ‐5D.

Three studies [12, 25, 27] compared the EQ‐5D of HRA and THA, indicating that there was no significant difference in EQ‐5D between HRA and THA [MD = −0.05, 95% CI = (−0.13, 0.02), *p* = 0.15], without heterogeneity (*p* = 0.76, *I* ^2^ = 0%). The results are shown in Figure 8.

#### 3.4.7 EQ‐5D VAS

**FIGURE 9:**
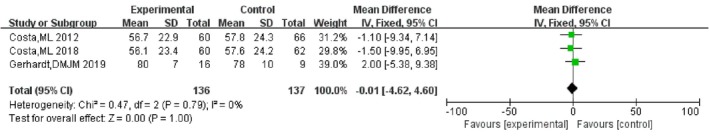
EQ‐5D VAS.

Three studies [12, 26, 27] compared the EQ‐5D VAS of HRA and THA, indicating that there was no significant difference in EQ‐5D VAS between HRA and THA [MD = −0.01, 95% CI = (−4.62, 4.60), *p* = 1.00], without heterogeneity (*p* = 0.79, *I* ^2^ = 0%). The results are shown in Figure 9.

#### 3.4.8 Blood Levels of Cobalt and Chromium

**FIGURE 10:**
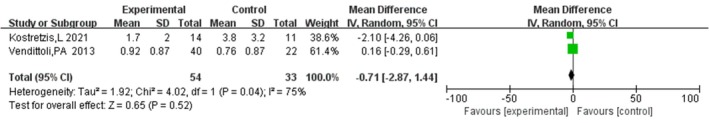
Blood levels of cobalt.

**FIGURE 11:**
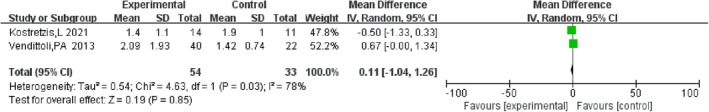
Blood levels of chromium.

Two studies [15, 19] compared the blood levels of cobalt and chromium of HRA and THA, indicating that there was no significant difference in the blood levels of cobalt and chromium between HRA and THA [cobalt: MD = −0.84, 95% CI = (−3.04, 1.36), *p* = 0.45; chromium: MD = 0.11, 95% CI = (−1.04, 1.26), *p* = 0.85], with high heterogeneity (cobalt: *p* = 0.45, *I* ^2^ = 88%; chromium: *p* = 0.85, *I* ^2^ = 90%). The results are shown in Figures 10 and 11.

#### 3.4.9 Complications

**FIGURE 12:**
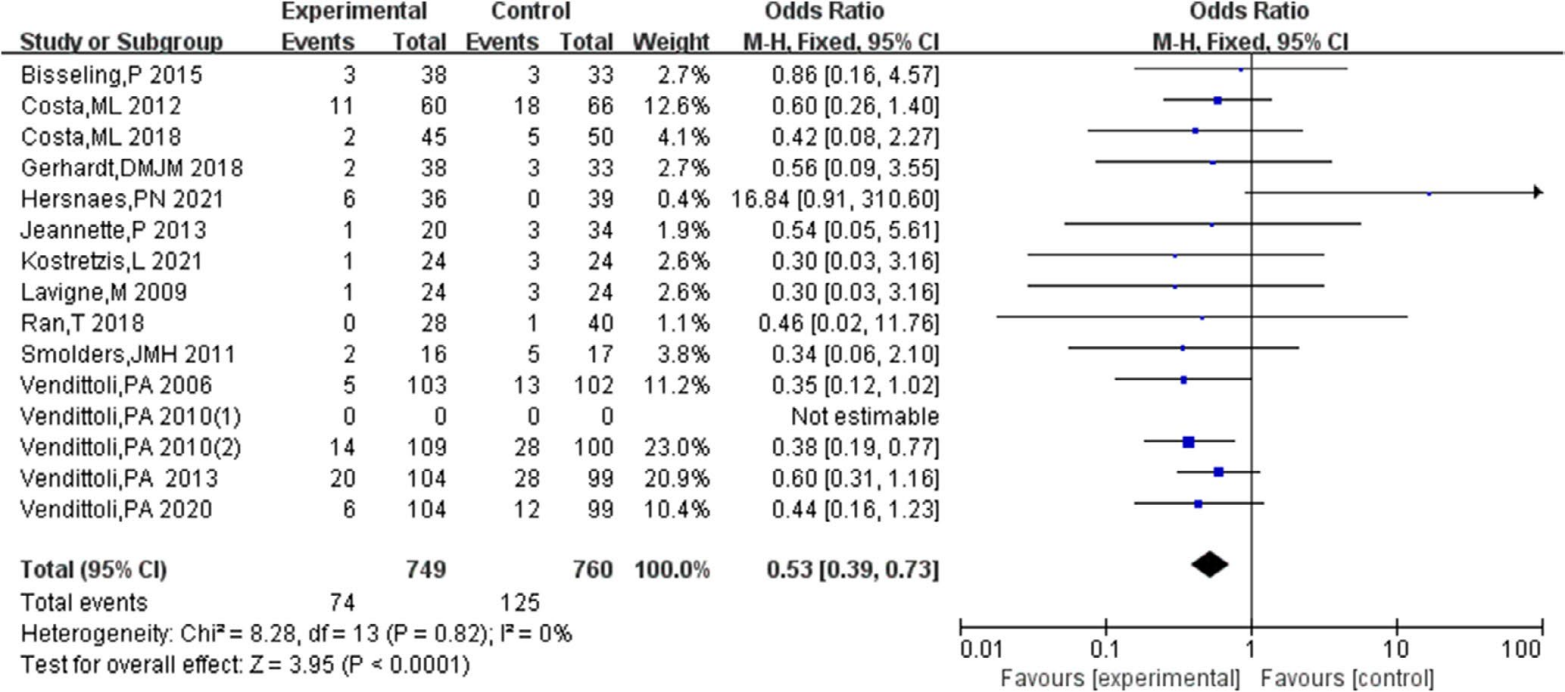
Complications.

Fifteen studies [11, 12, 13, 14, 15, 16, 17, 18, 19, 20, 22, 23, 25, 27, 29] compared the complications of HRA and THA, indicating that the complications of HRA were significantly lesser than those of THA [OR = 0.50, 95% CI = (0.39, 0.73), *p* < 0.00001], without heterogeneity (*p* = 0.74, *I* ^2^ = 0%). The results are shown in Figure 12.

#### 3.4.10 Blood Loss

**FIGURE 13:**
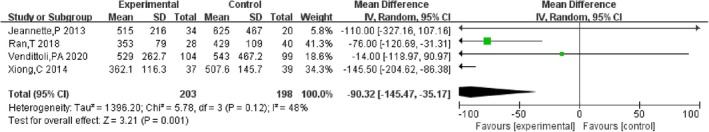
Blood loss.

Four studies [16, 20, 21, 25] compared the blood loss of HRA and THA, indicating that the blood loss of HRA was significantly less than that of THA [MD = −89.31, 95% CI = (−142.08, −36.55), *p* = 0.16], with low heterogeneity (*p* = 0.0009, *I* ^2^ *2* = 42%). The results are shown in Figure 13.

#### 3.4.11 Operating Time

**FIGURE 14:**
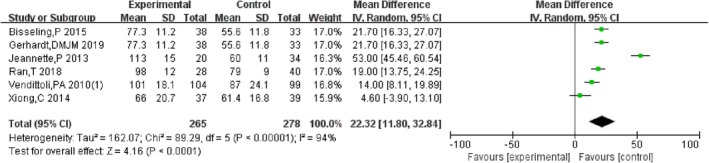
Operating time.

**FIGURE 15:**
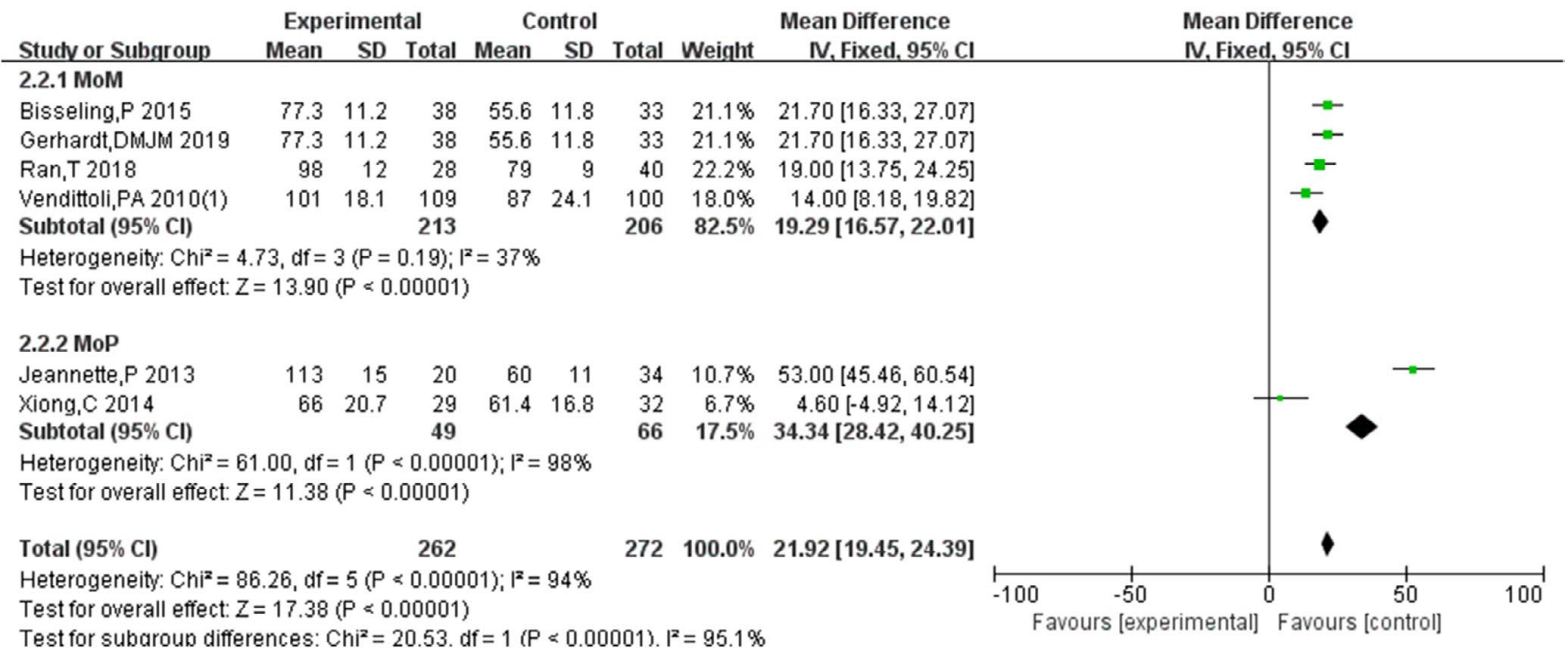
Operation time subgroup.

Six studies [11, 16, 21, 22, 25, 26] compared the operating time of HRA and THA, indicating that the operating time of HRA was significantly longer than that of THA [MD = 22.39, 95% CI = (11.00, 33.78), *p* = 0.0001], with high heterogeneity (*p* < 0.00001, *I* ^2^ = 94%). So, subgroup analysis was performed according to the type of prosthesis, and the result showed that compared to metal‐on‐metal THA, the operating time of HRA was significantly longer with low heterogeneity (*p* < 0.00001, *I* ^2^ = 37%) and compared to metal‐onon‐poly THA, the operating time of HRA was significantly longer with high heterogeneity (*p* < 0.00001, *I* ^2^ = 98%). The results are shown in Figures 14 and 15.

### 3.5 Publishing Bias Test

**FIGURE 16:**
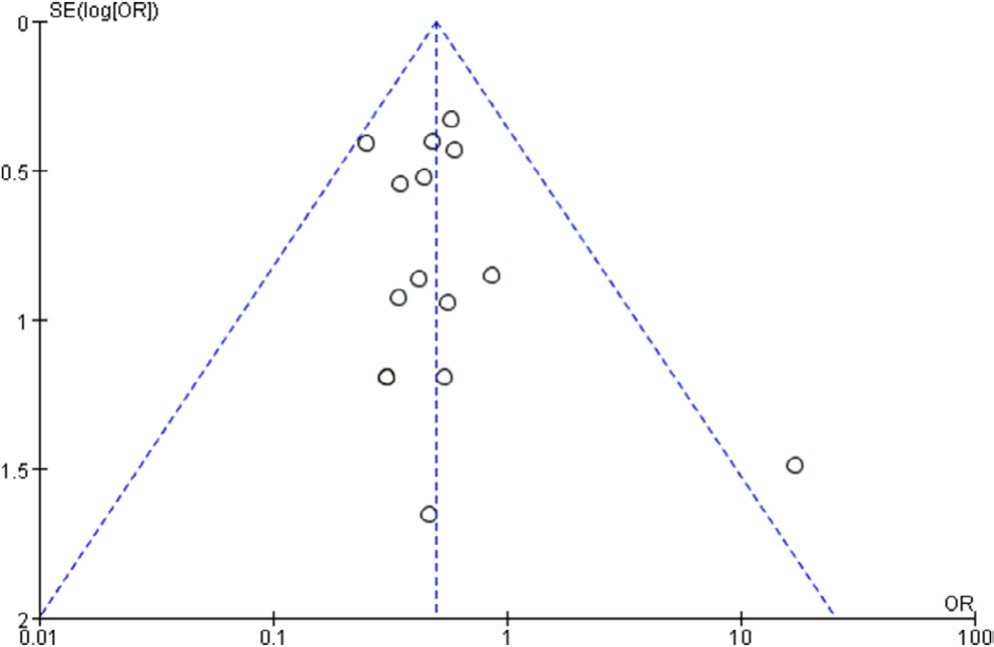
Complications funnel plot.

All outcomes were tested using a funnel plot. Taking the funnel plot of complications as an example, except for the study by Hersnaes, PN 2021, which deviates from the central axis, the overall distribution is symmetrical, indicating that there is no obvious bias in the included studies. However, in the study by Hersnaes, PN 2021, 16.7% (6 cases) and 15.4% (6 cases) of patients in the THA group and the HRA group were lost to follow‐up, respectively, which may lead to incomplete data and affect the accuracy of the results (Figure 16).

## 4 Discussion

### 4.1 Both HRA and THA Are Safe and Reliable Operations for End‐Stage Hip Arthrosis

Revision rate and complications are important indicators for assessing the safety, reliability, and survivorship of prostheses. In this meta‐analysis, loosening, periprosthetic fractures, and unexplained pain were the main complications of HAR, while dislocation, loosening, and infection were common after THA. The incidence of complications in the HRA group is significantly lower than in the THA group (9.88% vs. 16.44%). They may be due to the characteristics of HRA. HRA preserves more proximal femur bone and maintains the structural and biomechanical characteristics of the hip, which helps prevent dislocation and improve recovery of hip function. On the other hand, the insertion of the femoral component into the femoral neck in HRA can cause local stress concentration, closely related to femoral neck fractures post‐operation [30]. Therefore, precise surgical techniques for positioning the femoral component of HRA are critical for reducing complications.

It is well known that the survivorship of prostheses is influenced by complications notably. This meta‐analysis showed that although complications associated with HRA are significantly fewer than those of THA, the revision rates for HRA and THA are similar (6.45% vs. 5.3%), which is consistent with previous studies [31, 32]. The main reason for revision in HRA and THA is loosening and dislocation, respectively. Dislocation has been considered the most common cause of revision following THA, followed by periprosthetic joint infection and loosening [33]. Factors such as age, body mass index, surgical techniques, and femoral head size have been shown to influence the incidence of dislocation post‐THA [32, 34, 35]. Many studies [36, 37] have reported that males treated with HRA exhibit higher survivorship than females. The reasons may be related to differences in femoral head size and bone mineral density, which are generally lower in females. It has been confirmed that the revision rate for HRA decreases as femoral head size increases [38]. Factors such as gender, bone mineral density, femoral head size, material and type of prosthesis, and complications are crucial in influencing the need for revision and overall survivorship after HRA. Therefore, selecting ideal candidates for THA and HRA is critical. In this meta‐analysis, most included studies used large‐head prostheses for THA as the control group, which somewhat helped to reduce the revision rate post‐surgery, despite the lack of strict inclusion criteria for HRA. This might explain why HRA shows fewer complications, yet the revision rate is comparable to THA. Optimal candidate selection for HRA could potentially lower the revision rate even further. So, it is important to choose suitable patients and prevent prosthesis loosening to reduce the revision rate of HRA, and the results also indicate that HRA is a safe and reliable treatment for hip arthroplasty. Function scores are important for assessing the clinical effects of prostheses.

### 4.2 Both HRA and THA Are Effective for End‐Stage Hip Arthrosis

In this meta‐analysis, we used WOMAC, HSS, OHS, UCLA activity score, EQ‐5D, and ED‐5D VAS as primary outcomes to comprehensively assess the clinical effects of HRA and THA, including locomotion, daily activities, and pain. The results showed no difference in these function scores between HRA and THA, implicating that both procedures are similarly effective in restoring joint function and relieving pain after operation. Although some studies [39, 40] reported that HRA had potential advantages over THA in promoting patients to return to sport and high‐level activities early, and some studies [41] found that HRA had better function scores than those of THA, findings show that overall outcomes for HRA are comparable to THA. Additionally, because HRA preserves more bone of the femur and maintains hip joint morphology, being helpful in restoring native hip biomechanics and improving local stress, we think patients receiving HRA treatment will achieve better long‐term effects. Therefore, future prospective RCTs should focus on the long‐term effects of HRA and THA.

Metal wear after arthroplasty and debris have a close relationship with unexplained pain and adverse local tissue reactions [42]. Some scholars [43] reported a higher blood level of cobalt and chromium in patients with HRA compared to patients with THA. However, other studies indicate that blood levels of cobalt in patients treated by HRA were significantly lower than those of patients treated by MoM‐THA, with no significant differences in chromium levels. Theoretically, patients with THA should have higher debris and metal ion blood levels due to the junction of the femoral shaft and large femoral head (trunnion/taper junction), which may produce most of the metal ions, contributing to elevated levels of cobalt and chromium [44]. However, this meta‐analysis found no difference in blood levels of cobalt and chromium between HRA and THA. This discrepancy may be attributed to the high heterogeneity among the prostheses used in the included studies, such as metal‐on‐metal THA, metal‐on‐polyethylene THA, metal‐on‐metal HRA, which leads to high heterogeneity and influences the results of blood levels of cobalt and chromium. But among the included studies, there are only two studies reporting blood levels of cobalt and chromium. So, subgroup analysis is not available for further analysis. With the development of ceramic‐on‐ceramic and metal‐on‐polyethylene HRAs [45], the blood levels of cobalt and chromium in patients with HRA have the potential to decrease further. Currently, clinical outcomes from RCTs of typical ceramic‐on‐ceramic HRA like H1 [46] and ReCerf [47] have not been published. Besides, surgical technique, especially the position of the prosthesis, is an important factor influencing prosthesis wear and debris generation. In one of our pilot experiments about the biomechanical study of the location of the HRA prosthesis, neutral and varus positions of the femoral prosthesis in HRA may increase contact pressure, causing extra wear and debris, which may increase blood levels of cobalt and chromium. So, a valgus of 10°–15° of the femoral prosthesis may be an ideal position for HRA.

### 4.3 HRA and THA Have Their Own Advantages and Disadvantages in Terms of Surgical Techniques

Operating time and blood loss are secondary outcomes used to evaluate surgical techniques, which are related to approaches, surgical manners, skill, and experience of surgeons, etc. This meta‐analysis showed that compared to THA, HRA required more operating time but resulted in less blood loss, consistent with a previous study [48]. Although there is a high heterogeneity between studies reporting operating time, subgroup analysis according to the type of prosthesis showed that no matter compared to metal‐on‐metal THA or metal on poly THA, the operating time of HRA was longer and the main heterogeneity is related not only to the type of prosthesis but also to factors such as surgical techniques, the experience and cooperation of the medical team, individual characteristics of patients, and so on, in our opinion. On one hand, the surgical difficulty, accuracy, and learning curve of HRA are higher than those of THA, which may increase the operating time. On the other hand, since osteotomy of the femoral neck and enlargement of the medullary cavity are not needed during HRA, this may reduce blood loss. Thus, both HRA and THA have their own advantages and disadvantages in terms of surgical techniques.

### 4.4 Strengths and Limitations

Although some studies [41, 48, 49] have conducted comparative analyses of HRA and THA, this meta‐analysis includes the largest number of RCTs, which are from six countries, and evaluates 11 outcomes. The results reveal that compared to THA, HRA has a similar revision rate, functional scores, and blood levels of cobalt and chromium. Additionally, HRA results in fewer complications and less blood loss, indicating its effectiveness and safety as a treatment option for hip replacement.

Obviously, there also are some limitations in the study. (i) Some results, especially operating time and blood levels of cobalt and chromium, show high heterogeneity among included studies, which cannot be resolved by subgroup analysis because there are only two papers reporting this result. This affects the reliability and accuracy of results. (ii) The follow‐up period for most included studies is short, requiring more prospective RCTs with long‐time follow‐up to analyze the long‐term effects of HRA compared to THA. (iii) No included studies have used ceramic‐on‐ceramic HRA; thus, additional prospective RCTs analyzing HRA are needed in the future.

### 4.5 Prospects of Clinical Application

For patients with end‐stage hip arthrosis, especially active men under 60 years of age and male manual laborers for whom squatting is often required but typically restricted after THA, HRA is a safe and effective alternative.

## 5 Conclusion

Both HRA and THA are safe and effective for end‐stage hip arthrosis with similar revision rates, complications incidence, and effects on functional recovery. Considering the advantages of HRA in minimally invasive procedures and preserving more bone, it is particularly suited for active men under 60 years of age and male manual laborers, for whom squatting is often required but typically restricted after THA.

## Author Contributions

X.F. and Y.Z. contributed equally to this work. J.P.C. and T.Y. took part in the design of the study. X.F. and Y.Z. performed the literature survey, and drafted the manuscript. T.Z. took part in the data management implementation of the study. All authors contributed to the article and approved the submitted version.

## Conflicts of Interest

The authors declare no conflicts of interest.

## Data Availability

The authors have nothing to report.

## References

[1] L. Okafor and A. F. Chen , “Patient Satisfaction and Total Hip Arthroplasty: A Review,” Arthroplasty 1, no. 1 (2019): 6. 35240763 10.1186/s42836-019-0007-3 PMC8787874

[2] M. A. Alagha , K. Logishetty , C. O'Hanlon , A. D. Liddle , and J. Cobb , “Three‐Dimensional Preoperative Planning Software for Hip Resurfacing Arthroplasty,” Bioengineering 10, no. 8 (2023): 939, 10.3390/bioengineering10080939. 37627824 PMC10451941

[3] T. Al‐Jabri , M. Ridha , R. A. McCulloch , et al., “Hip Resurfacing Arthroplasty: Past, Present and Future,” Orthopedic Reviews 15 (2023): 77745, 10.52965/001c.77745. 37405271 PMC10317512

[4] D. G. LeBrun , T. S. Shen , P. Bovonratwet , R. Morgenstern , and E. P. Su , “Hip Resurfacing vs Total Hip Arthroplasty in Patients Younger Than 35 Years: A Comparison of Revision Rates and Patient‐Reported Outcomes,” Arthroplast Today 11 (2021): 229–233. 34692960 10.1016/j.artd.2021.09.004 PMC8516816

[5] K. Rueckl , A. Liebich , U. Bechler , B. Springer , M. Rudert , and F. Boettner , “Return to Sports After Hip Resurfacing Versus Total Hip Arthroplasty: A Mid‐Term Case Control Study,” Archives of Orthopaedic and Trauma Surgery 140, no. 7 (2020): 957–962. 32296965 10.1007/s00402-020-03414-6

[6] S. Konan , C. Waugh , N. Ohly , C. P. Duncan , B. A. Masri , and D. S. Garbuz , “Mid‐Term Results of a Prospective Randomised Controlled Trial Comparing Large‐Head Metal‐on‐Metal Hip Replacement to Hip Resurfacing Using Patient‐Reported Outcome Measures and Objective Functional Task‐Based Outcomes,” Hip International 31, no. 5 (2021): 637–643. 32390475 10.1177/1120700020919671

[7] J. O. Penny , K. Brixen , J. E. Varmarken , O. Ovesen , and S. Overgaard , “Changes in Bone Mineral Density of the Acetabulum, Femoral Neck and Femoral Shaft, After Hip Resurfacing and Total Hip Replacement: Two‐Year Results From a Randomised Study,” Journal of Bone and Joint Surgery. British Volume 94, no. 8 (2012): 1036–1044. 22844043 10.1302/0301-620X.94B8.28222

[8] L. Savarino , M. Cadossi , E. Chiarello , N. Baldini , and S. Giannini , “Do Ion Levels in Metal‐on‐Metal Hip Resurfacing Differ From Those in Metal‐on‐Metal THA at Long‐Term Followup?,” Clinical Orthopaedics and Related Research 471, no. 9 (2013): 2964–2971. 23572350 10.1007/s11999-013-2981-z PMC3734417

[9] G. H. Prosser , P. J. Yates , D. J. Wood , S. E. Graves , R. N. de Steiger , and L. N. Miller , “Outcome of Primary Resurfacing Hip Replacement: Evaluation of Risk Factors for Early Revision,” Acta Orthopaedica 81, no. 1 (2010): 66–71. 20180719 10.3109/17453671003685434 PMC2856206

[10] M. Cumpston , T. Li , M. J. Page , et al., “Updated Guidance for Trusted Systematic Reviews: A New Edition of the Cochrane Handbook for Systematic Reviews of Interventions,” Cochrane Database of Systematic Reviews 10, no. 10 (2019): Ed000142, 10.1002/14651858.ED000142. 31643080 PMC10284251

[11] P. Bisseling , J. M. Smolders , A. Hol , and J. L. van Susante , “Metal Ion Levels and Functional Results Following Resurfacing Hip Arthroplasty Versus Conventional Small‐Diameter Metal‐on‐Metal Total Hip Arthroplasty; A 3 to 5year Follow‐Up of a Randomized Controlled Trial,” Journal of Arthroplasty 30, no. 1 (2015): 61–67. 25172584 10.1016/j.arth.2014.07.036

[12] M. L. Costa , J. Achten , P. Foguet , and N. R. Parsons , “Comparison of Hip Function and Quality of Life of Total Hip Arthroplasty and Resurfacing Arthroplasty in the Treatment of Young Patients With Arthritis of the Hip Joint at 5 Years,” BMJ Open 8, no. 3 (2018): e018849. 10.1136/bmjopen-2017-018849 PMC5879574 29530907

[13] D. M. Gerhardt , J. M. Smolders , E. A. Roovers , T. A. Rijnders , and J. L. van Susante , “Changes in Periacetabular Bone Mineral Density Five Years After Resurfacing Hip Arthroplasty Versus Conventional Total Hip Arthroplasty,” Hip International 29, no. 2 (2018): 153–160. 30426791 10.1177/1120700018808023

[14] P. N. Hersnaes , K. Gromov , K. S. Otte , P. H. Gebuhr , and A. Troelsen , “Harris Hip Score and SF‐36 Following Metal‐on‐Metal Total Hip Arthroplasty and Hip Resurfacing ‐ a Randomized Controlled Trial With 5‐Years Follow Up Including 75 Patients,” BMC Musculoskeletal Disorders 22, no. 1 (2021): 781. 34511090 10.1186/s12891-021-04671-1 PMC8436430

[15] L. Kostretzis , M. Lavigne , M. O. Kiss , M. Shahin , J. Barry , and P. A. Vendittoli , “Despite Higher Revision Rate, MoM Large‐Head THA Offers Better Clinical Scores Than HR: 14‐Year Results From a Randomized Controlled Trial Involving 48 Patients,” BMC Musculoskeletal Disorders 22, no. 1 (2021): 400. 33941155 10.1186/s12891-021-04286-6 PMC8091753

[16] R. Tao , F. Liu , Y. K. Liu , et al., “A Prospective Comparative Study of Hip Resurfacing Arthroplasty and Large‐Diameter Head Metal‐on‐Metal Total Hip Arthroplasty in Younger Patients‐a Minimum of Five Year Follow‐Up,” International Orthopaedics 42, no. 10 (2018): 2323–2327. 29455347 10.1007/s00264-018-3819-9

[17] J. M. Smolders , A. Hol , W. J. Rijnberg , and J. L. van Susante , “Metal Ion Levels and Functional Results After Either Resurfacing Hip Arthroplasty or Conventional Metal‐on‐Metal Hip Arthroplasty,” Acta Orthopaedica 82, no. 5 (2011): 559–566. 22103280 10.3109/17453674.2011.625533 PMC3242952

[18] P. A. Vendittoli , A. Roy , S. Mottard , J. Girard , D. Lusignan , and M. Lavigne , “Metal Ion Release From Bearing Wear and Corrosion With 28 mm and Large‐Diameter Metal‐on‐Metal Bearing Articulations: A Follow‐Up Study,” Journal of Bone and Joint Surgery. British Volume 92, no. 1 (2010): 12–19. 20044673 10.1302/0301-620X.92B1.22226

[19] P. A. Vendittoli , C. Rivière , A. G. Roy , J. Barry , D. Lusignan , and M. Lavigne , “Metal‐on‐Metal Hip Resurfacing Compared With 28‐mm Diameter Metal‐on‐Metal Total Hip Replacement: A Randomised Study With Six to Nine years' Follow‐Up,” Bone & Joint Journal 95‐b, no. 11 (2013): 1464–1473. 10.1302/0301-620X.95B11.31604 24151264

[20] P. A. Vendittoli , M. Shahin , C. Rivière , A. G. Roy , J. Barry , and M. Lavigne , “Hip Resurfacing Compared With 28‐mm Metal‐on‐Metal Total Hip Replacement: A Randomized Study With 15 Years of Follow‐Up,” Journal of Bone and Joint Surgery. American Volume 102, no. Suppl 2 (2020): 80–90. 32554999 10.2106/JBJS.20.00030

[21] C. Xiong , Comparative Study on Clinical Efficacy Between Hip Resurfacing Arthroplasty and Total Hip Arthroplasty (Tianjin Medical University, 2014).

[22] P. A. Vendittoli , M. Ganapathi , A. G. Roy , D. Lusignan , and M. Lavigne , “A Comparison of Clinical Results of Hip Resurfacing Arthroplasty and 28 mm Metal on Metal Total Hip Arthroplasty: A Randomised Trial With 3‐6 Years Follow‐Up,” Hip International 20, no. 1 (2010): 1–13. 20235065 10.1177/112070001002000101

[23] M. Lavigne , M. Therrien , J. Nantel , A. Roy , F. Prince , and P. A. Vendittoli , “The John Charnley Award: The Functional Outcome of Hip Resurfacing and Large‐Head THA Is the Same: A Randomized, Double‐Blind Study,” Clinical Orthopaedics and Related Research 468, no. 2 (2010): 326–336. 19543863 10.1007/s11999-009-0938-z PMC2807020

[24] P. K. Karampinas , D. S. Evangelopoulos , J. Vlamis , K. Nikolopoulos , and D. S. Korres , “Confronting Hip Resurfacing and Big Femoral Head Replacement Gait Analysis,” Orthopedic Reviews 6, no. 1 (2014): 5221. 24744841 10.4081/or.2014.5221 PMC3980157

[25] J. Penny , O. Ovesen , J. E. Varmarken , and S. Overgaard , “Similar Range of Motion and Function After Resurfacing Large‐Head or Standard Total Hip Arthroplasty,” Acta Orthopaedica 84, no. 3 (2013): 246–253. 23530872 10.3109/17453674.2013.788435 PMC3715815

[26] D. Gerhardt , T. G. T. Mors , G. Hannink , and J. L. C. Van Susante , “Resurfacing Hip Arthroplasty Better Preserves a Normal Gait Pattern at Increasing Walking Speeds Compared to Total Hip Arthroplasty,” Acta Orthopaedica 90, no. 3 (2019): 231–236. 30931667 10.1080/17453674.2019.1594096 PMC6534262

[27] M. L. Costa , J. Achten , N. R. Parsons , et al., “Total Hip Arthroplasty Versus Resurfacing Arthroplasty in the Treatment of Patients With Arthritis of the Hip Joint: Single Centre, Parallel Group, Assessor Blinded, Randomised Controlled Trial,” BMJ 344, no. apr19 1 (2012): e2147, 10.1136/bmj.e2147. 22517930 PMC3330050

[28] L. X. Wang , “Total Hip Arthroplasty or Total Hip Resurfacing Arthroplasty for 32 Cases of Secondary Post‐Traumatic Arthritis After Internal Fixation of Acetabular Fractures,” Shandong Medical Journal 55, no. 47 (2015): 88–89.

[29] P. A. Vendittoli , M. Lavigne , A. G. Roy , and D. Lusignan , “A Prospective Randomized Clinical Trial Comparing Metal‐on‐Metal Total Hip Arthroplasty and Metal‐on‐Metal Total Hip Resurfacing in Patients Less Than 65 Years Old,” Hip International 16 (2006): S73–S81. 10.1177/112070000601604S14 19219833

[30] D. Vogel , M. Wehmeyer , M. Kebbach , H. Heyer , and R. Bader , “Stress and Strain Distribution in Femoral Heads for Hip Resurfacing Arthroplasty With Different Materials: A Finite Element Analysis,” Journal of the Mechanical Behavior of Biomedical Materials 113 (2021): 104115. 33189013 10.1016/j.jmbbm.2020.104115

[31] H. Subbiah Ponniah , K. Logishetty , T. C. Edwards , and G. C. Singer , “Survivorship and Risk Factors for Revision of Metal‐on‐Metal Hip Resurfacing,” Bone & Joint Open 4, no. 11 (2023): 853–858. 37944559 10.1302/2633-1462.411.BJO-2023-0084.R1 PMC10635743

[32] O. Pakarinen , O. Lainiala , A. Reito , P. Neuvonen , K. Mäkelä , and A. Eskelinen , “Implant Survival of 662 Dual‐Mobility Cups and 727 Constrained Liners in Primary THA: Small Femoral Head Size Increases the Cumulative Incidence of Revision,” Acta Orthopaedica 92, no. 6 (2021): 658–664. 34238130 10.1080/17453674.2021.1939597 PMC8641668

[33] W. Hoskins , S. Rainbird , M. Lorimer , S. E. Graves , and R. Bingham , “What Can we Learn From Surgeons Who Perform THA and TKA and Have the Lowest Revision Rates? A Study From the Australian Orthopaedic Association National Joint Replacement Registry,” Clinical Orthopaedics and Related Research 480, no. 3 (2022): 464–481. 34677162 10.1097/CORR.0000000000002007 PMC8846272

[34] F. E. Rowan , B. Benjamin , J. R. Pietrak , and F. S. Haddad , “Prevention of Dislocation After Total Hip Arthroplasty,” Journal of Arthroplasty 33, no. 5 (2018): 1316–1324. 29525344 10.1016/j.arth.2018.01.047

[35] P. J. Duwelius , R. D. Southgate , J. P. Crutcher, Jr. , et al., “Registry Data Show Complication Rates and Cost in Revision Hip Arthroplasty,” Journal of Arthroplasty 38, no. 7s (2023): S29–S33, 10.1016/j.arth.2023.04.050. 37121489

[36] J. Stoney , S. E. Graves , R. N. de Steiger , S. Rainbird , T. L. Kelly , and A. Hatton , “Is the Survivorship of Birmingham Hip Resurfacing Better Than Selected Conventional Hip Arthroplasties in Men Younger Than 65 Years of Age? A Study From the Australian Orthopaedic Association National Joint Replacement Registry,” Clinical Orthopaedics and Related Research 478, no. 11 (2020): 2625–2636. 32898048 10.1097/CORR.0000000000001453 PMC7571983

[37] M. Q. Azam , S. McMahon , G. Hawdon , and S. R. Sankineani , “Survivorship and Clinical Outcome of Birmingham Hip Resurfacing: A Minimum Ten Years' Follow‐Up,” International Orthopaedics 40, no. 1 (2016): 1–7. 25820838 10.1007/s00264-015-2731-9

[38] G. S. Donahue , V. Lindgren , V. P. Galea , R. Madanat , O. Muratoglu , and H. Malchau , “Are Females at Greater Risk for Revision Surgery After Hip Resurfacing Arthroplasty With the Articular Surface Replacement Prosthesis?,” Clinical Orthopaedics and Related Research 474, no. 10 (2016): 2257–2265. 27121872 10.1007/s11999-016-4860-x PMC5014806

[39] M. D. Hellman , M. C. Ford , and R. L. Barrack , “Is There Evidence to Support an Indication for Surface Replacement Arthroplasty?: A Systematic Review,” Bone & Joint Journal 101‐b, no. 1_Supple_A (2019): 32–40. 10.1302/0301-620X.101B1.BJJ-2018-0508.R1 30648490

[40] A. Magan , W. Wignadasan , B. Kayani , G. Radhakrishnan , F. Ronca , and F. S. Haddad , “A Meta‐Analysis Assessing Time for Return to Sport Following Hip Resurfacing,” Archives of Orthopaedic and Trauma Surgery 143, no. 6 (2023): 3575–3585. 36040530 10.1007/s00402-022-04592-1

[41] P. Kumar , V. Ksheersagar , S. Aggarwal , et al., “Complications and Mid to Long Term Outcomes for Hip Resurfacing Versus Total Hip Replacement: A Systematic Review and Meta‐Analysis,” European Journal of Orthopaedic Surgery & Traumatology: Orthopedie Traumatologie 33, no. 5 (2023): 1495–1504, 10.1007/s00590-022-03361-5. 36006506

[42] V. P. Galea , I. Laaksonen , J. W. Connelly , et al., “What Is the Clinical Presentation of Adverse Local Tissue Reaction in Metal‐on‐Metal Hip Arthroplasty? An MRI Study,” Clinical Orthopaedics and Related Research 477, no. 2 (2019): 353–360, 10.1097/CORR.0000000000000393. 30794223 PMC6370095

[43] M. F. Koff , M. A. Gao , J. P. Neri , et al., “Adverse Local Tissue Reactions Are Common in Asymptomatic Individuals After Hip Resurfacing Arthroplasty: Interim Report From a Prospective Longitudinal Study,” Clinical Orthopaedics and Related Research 479, no. 12 (2021): 2633–2650, 10.1097/CORR.0000000000001882. 34232144 PMC8726542

[44] M. J. Taunton , “How to Interpret Metal Ions in THA,” Journal of Arthroplasty 35, no. 6s (2020): S60–S62. 10.1016/j.arth.2020.01.010 32061479

[45] D. de Villiers and S. Collins , “Wear of Large Diameter Ceramic‐on‐Ceramic Hip Bearings Under Standard and Microseparation Conditions,” Biotribology 21 (2020): 100117.

[46] A. Maslivec , C. Halewood , S. Clarke , and J. Cobb , “Hip Resurfacing Arthroplasty in Women: A Novel Ceramic Device Enables Near Normal Gait Function,” Gait & Posture 103 (2023): 166–171. 37210849 10.1016/j.gaitpost.2023.05.015

[47] D. de Villiers and S. Collins , “Resistance of a Novel Ceramic Acetabular Cup to Critical Impact Loads,” Proceedings of the Institution of Mechanical Engineers. Part H 234, no. 10 (2020): 1122–1128. 10.1177/0954411920941383 32633704

[48] M. Palazzuolo , A. Bensa , S. Bauer , W. G. Blakeney , G. Filardo , and M. Riegger , “Resurfacing Hip Arthroplasty Is a Safe and Effective Alternative to Total Hip Arthroplasty in Young Patients: A Systematic Review and Meta‐Analysis,” Journal of Clinical Medicine 12, no. 6 (2023): 2093. 36983096 10.3390/jcm12062093 PMC10052473

[49] D. A. Marshall , K. Pykerman , J. Werle , et al., “Hip Resurfacing Versus Total Hip Arthroplasty: A Systematic Review Comparing Standardized Outcomes,” Clinical Orthopaedics and Related Research 472, no. 7 (2014): 2217–2230. 24700446 10.1007/s11999-014-3556-3 PMC4048407

